# Changes in Sexuality and Quality of Couple Relationship During the COVID-19 Lockdown

**DOI:** 10.3389/fpsyg.2020.565823

**Published:** 2020-09-29

**Authors:** Marta Panzeri, Roberta Ferrucci, Angela Cozza, Lilybeth Fontanesi

**Affiliations:** ^1^Department of Developmental Psychology and Socialisation, Padua University, Padua, Italy; ^2^Aldo Ravelli Center, University of Milan, Milan, Italy; ^3^San Paolo Hospital, Milan, Italy; ^4^Department of Psychological Health and Territorial Science, University G. D’Annunzio Chieti-Pescara, Chieti, Italy

**Keywords:** sexuality, COVID-19, couples, anxiety, fear, psychological distress

## Abstract

The COVID-19 pandemic is heavily influencing people’s general well-being worldwide. Since its outbreak, many studies have explored the population’s general psychological well-being, while only a few studies have addressed how the COVID-19 pandemic and the lockdown are affecting sexuality. Sexual health, an important aspect of general well-being, has relevant consequences on people’s daily lives. Although it is well known that distress can affect sexuality, and it is possible to speculate that the outbreak’s psychological outcomes are affecting the population’s sexual life; recent literature does not explore couples’ sexuality and their relationship quality during the lockdown. The present preliminary research aimed to understand if the Italian population’s sexuality has changed, and if so, how it had changed since the spread of COVID-19, and which variables were influencing couples’ relationship quality during the COVID-19 lockdown. A questionnaire reserved especially for cohabiting couples was designed and distributed online from April 11 to May 5, 2020, the 5th and 8th weeks, respectively, after the start of the lockdown. Of the 124 respondents who completed the online survey, 73% were females. Despite the pandemic’s psychological consequences, when asked directly, most couples responded that they did not perceive any differences in their sexuality. However, some female participants did report a decrease in pleasure, satisfaction, desire, and arousal. The main reasons behind the changes in sexuality in women, therefore, appear to be worry, lack of privacy, and stress. Even when participants seemed to show high levels of resilience, the negative aspects of lockdown could affect their quality of sexual life. This study needs to be completed using qualitative data from online focus groups that have investigated how sexual life has changed and the main needs of couples. All the same, our results will serve to better address population needs and experiences, and provide *ad hoc* interventions during this unprecedented time of crisis.

## Introduction

The COVID-19 pandemic is highly affecting people’s general well-being worldwide (Cao et al., 2020; Wang et al., 2020; White and Van Der Boor, 2020). Recent literature has shown that the uncertainties about health and work, combined with social distancing and homeschooling connected to the forced lockdown, have had an impact on psychological adjustment, influencing anxiety and depression levels, sleep and eating patterns, and somatic symptomatology (Ahmed et al., 2020; Cellini et al., 2020; Fernández-Aranda et al., 2020; Huang and Zhao, 2020; Tian et al., 2020; Zhang et al., 2020). Negative emotional responses have been found both in the general adult population and medical care staff, as well as in children and adolescents, especially after the announcement of the COVID-19 pandemic. This is coherent with previous research that found that such public emergencies concerning health usually trigger a series of stressful emotional responses characterized by high levels of anxiety and generally negative emotions, along with a decrease in positive feelings during the same time period (Brooks et al., 2020; Li S. et al., 2020). Similar or the same results were found in Italy: depressive symptoms, anxiety, and stress were increased in women, people between 30 and 34 years, and people with previous medical problems (Ferrucci et al., 2020; Mazza et al., 2020; Rossi et al., 2020); sleep quality has worsened, especially for those living in Northern Italy, the most affected by COVID-19 (Casagrande et al., 2020; Rossi et al., 2020); the psychological impact of the lockdown was much higher in northern regions than in the central-southern ones, and the most frequent fears were those about the economic crisis, getting the infection, and dying (Ferrucci et al., 2020). While many studies are exploring the population’s general psychological well-being, a few studies are addressing how COVID-19 and the lockdown are affecting sexuality. Sexual health is an important aspect of general well-being, with important consequences on the population’s daily lives (Ford et al., 2019), from different points of view. In particular, it is not clear whether sexual behaviors among married couples have changed during the lockdown (Arafat et al., 2020), as well as the role played by psychological and personal variables, in this process. Due to these reasons, this preliminary study aims at understanding the main changes that people are facing in their sexual lives as well as identifying the main core issues based on the online focus groups’ incoming data on sexual well-being during this period of crisis. While it is well known that distress can impair sexuality (Montesi et al., 2013; Leavitt and Willoughby, 2015; Rokach, 2019), and it is possible to speculate that at present, the psychological outcomes of COVID-19 are affecting the population’s sexual life, recent literature does not explore couple sexuality and the quality of relationships during the lockdown. In Italy, for example, when the government decided on a sudden lockdown during the second week of March, some couples were separated, while others were forced to live together in the same home. Besides, due to the pandemic’s psychological outcomes, some couples’ daily personal lives have been drastically overturned. On the one hand, partners have lost their privacy due to the constant presence of children or other family members, whereas on the other hand, forced lockdown can exacerbate existing relationship problems. Fear of being infected also drastically reduced physical contact in couples: a decrease in vaginal sex was found in United States couples (Hensel et al., 2020), while in the United Kingdom, 60.1% of the participants did not engage in sexual activity during the self-isolation period, while the remaining 39.9% had sex at least once a week, where being male, young, and married was associated with an increase in sexual activity, and a prolonged period of quarantine was associated with an increase in sexual activity, probably due to reduced stress and anxiety or as a diversion to deal with boring days (Jacob et al., 2020). In another study, 43.5% of the participants from several countries reported a decline in sexual quality along with a severe reduction in the frequency of intercourse during lockdown compared to the previous year, even if many people living with the partner have experimented new sexual positions, BDSM, and acted out some sexual fantasies, while those who did not live with the partner tried new activities such as sexting (Lehmiller et al., 2020).

The problem of forced prolonged cohabitation has an impact not only on couple sexuality but also on autoerotic sexuality, which comprises an important aspect of self-regulation and sexual well-being. A recent online survey conducted between March and April 2020 in both England and Spain reported that 10% of the participants masturbated more than usual during quarantine (Ibarra et al., 2020). In another study conducted online in China from May 1–10, 2020, 30% of the participants declared an increase in masturbation and pornography use (Li G. et al., 2020). During the lockdown period, PornHub noticed a worldwide increase in pornography; for example, in the states where PornHub gave free access to its premium services, the increase observed was 57% in Italy, 38% in France, and 61% in Spain (Pornhub Insights, n.d.). Similar patterns were also found in the United States and some Asian jurisdictions (Mestre-Bach et al., 2020). A possible explanation for this increase in the use of pornographic material during the lockdown could be trying to manage the stress due to the changes in daily life that occurred during the quarantine and a short-term method of relief or to compensate the sense of loneliness (Uzieblo and Prescott, 2020). Autoerotic and dyadic sexuality, which play a significant role in sexual self-regulation, especially during crisis situations such as the COVID-19 pandemic should attract the attention of professionals because very few studies have assessed the quality of sexual life after a prolonged period of both forced cohabitation and social distancing.

In the light of these considerations, the present preliminary research aimed to understand if the Italian population’s sexuality had changed since the spread of the COVID-19 infection, and if so, how it had changed, as well as to examine the variables influencing the couples’ relationship quality during the COVID-19 lockdown.

## Materials and Methods

### Participants and Procedure

Participants (124; 73.4% female) took part in the survey, aged between 23 and 60 years old (*M* = 34.01, SD = 8.71). The inclusion criteria comprised being at least 18 years old and living with a partner during the lockdown, and speak and understand the Italian language. The characteristics of the sample are reported in Table 1.

**TABLE 1:** Demographic characteristic of the sample.

	*N*	%
**Gender**		
Male	33	26.6
Female	91	73.4
**Age category**		
≤34	75	61.5
35–50	38	31.1
≥50	9	7.4
**Living with**		
Only partner	72	58.1
Partner and children	41	33.1
Partner and other family members	6	4.8
Partner and other people	5	4.0
**Living in**		
North Italy	96	77.4
Center Italy	6	4.8
South Italy	17	13.7
**Work**		
Do not work	46	37.4
Outside the home	28	22.8
Partly at home, partly outside	12	9.8
At home	37	30.1
**Sexual orientation**		
Heterosexual	116	94.4
Bisexual	5	4.0
Homosexual	2	1.6

The questionnaire was designed for online completion to expedite data gathering during the COVID-19 lockdown. People were invited to take part in the study via social media. The procedure involved agreeing to an online consent form. All data were confidential and were stored in a password-protected electronic format.

The questionnaire link was sent to individuals as well as to associations and clubs through Facebook and Whatsapp groups, with a request to forward the information within their groups, nationally. The initial invitation to participate was sent on April 11, 2020 (the 5th week of the lockdown). The survey remained open until May 5, 2020 (the 8th week of the lockdown), and the date of completion was recorded with each respondent’s data. This period was chosen to study the effects of the lockdown starting from 1 month after the beginning of the lockdown and ending when restrictions began to loosen. The protocol for this study was reviewed and approved by the ethics committee of the Psychological Research Area 17 of Padua University.

### Measures

Participants were administered a demographic and part of five standardized questionnaires, along with a set of *ad hoc* questions investigating possible changes in their sexual life during the lockdown as well as the reasons for these changes, to give them the freedom to express their beliefs and opinions.

With regard to the Brief Index of Sexual Functioning for Women (BISF-W) (Taylor et al., 1994), its Italian validation (Panzeri et al., 2009), and the Italian version of the Brief Index of Sexual Functioning for Men (BISF-M) (Panzeri and Raoli, 2010), we used two factors: couple sexuality (21 items, alpha = 0.95 for women and alpha = 0.94 for men) and autoeroticism (6 items, alpha = 0.85 for women and alpha = 0.89 for men) plus 14 items that explored sexual satisfaction and sexual problems. Items were arranged in a Likert-type format, ranging from 5 to 7 points, to rate the frequency of the occurrence of sexual desires, arousal, orgasm, or satisfaction.

We used two factors of the Sexual Desire Inventory (SDI) (Spector et al., 1996): dyadic sexual desire (six items, alpha = 0.80 for women and alpha = 0.80 for men) and solitaire sexual desire (four items, alpha = 0.88 for women and alpha = 0.93 for men) (Moyano et al., 2017).

We used the three factors of the Depression Anxiety Stress Scales-21 (DASS-21) (Henry and Crawford, 2005; Italian validation by Bottesi et al., 2015): depression (7 items, alpha = 0.82), anxiety (7 items, alpha = 0.74), stress (7 items, alpha = 0.85), and the total score (21 items, alpha = 0.90).

The total score of the Patient Health Questionnaire (PHQ-15) (Kroenke et al., 2002) was used to assess 15 somatic symptoms. Each symptom was scored from 0 (“not bothered at all”) to 2 (“bothered a lot”). Cronbach’s alpha for the present research was alpha = 0.72.

The Quality of Marriage Index (QMI) (Norton, 1983) is a six-item measure of global perceptions of couple relationship satisfaction, in which higher scores indicate higher levels of satisfaction (alpha = 0.96).

### Statistical Analysis

Descriptive statistics were used to calculate categorical variables. The percentage of responses was calculated according to the number of responses in each category divided by the total number of respondents to a question. A cluster analysis, using TwoStep cluster analysis for binary data, was performed to divide the sample according to the changes in their sexuality (desire, frequency, satisfaction, orgasm, pleasure, excitation, and importance). The analysis returned two groups, based on whether sexual life has been changed during the COVID-19 lockdown, allowing *t*-test analysis and Chi-square analysis to assess the differences in the study variables between the two groups.

We performed logistic regression using the SPSS program version 25 with age, DASS factors, as well as the total score, BISF factors, QMI, and PHQ as the independent variables, and sexuality changes and quality of marriage as the dependent variables.

## Results

### Changes in Sexuality During the COVID-19 Lockdown

**FIGURE 1:**
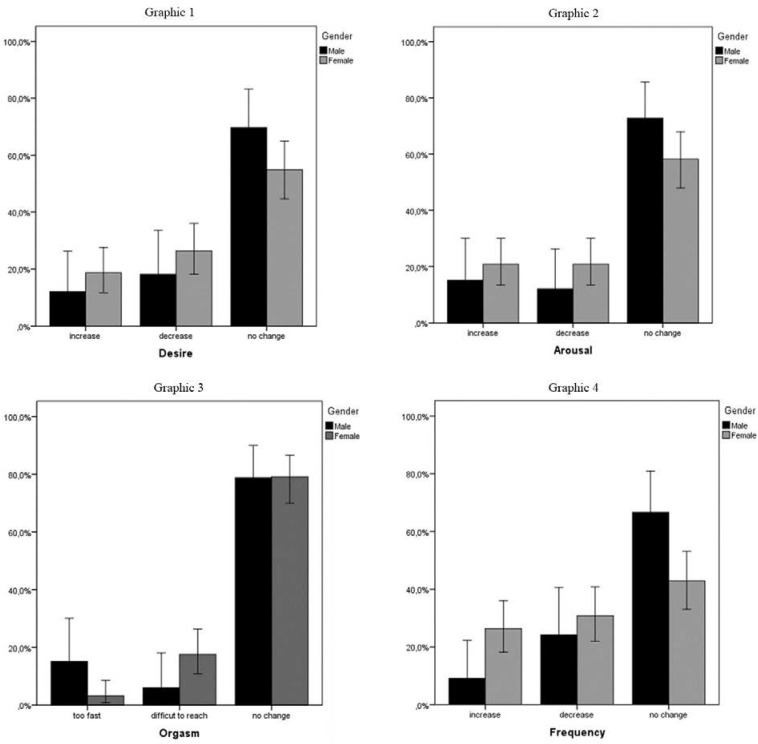
Change in sexual desire, sexual arousal, orgasm, and sexual activity frequency.

Among the participants, 12.1% men and 18.7% women perceived an increase in sexual desire during the lockdown, while 18.2% men and 26.4% women perceived a decrease in sexual desire (Figure 1). Men (15.2%) and women (20.9%) observed an increase in arousal during the lockdown, while 12.1% men and 20.9% women observed a decrease in arousal during the same period. Moreover, the women experienced more difficulty in reaching orgasm than the men (6.1% men, 17.6% women), while in comparison with the pre-lockdown period, men reported reaching orgasms faster and more frequently than women (15.2% men, 3.3% women).

In relation to the three main areas of research (sexual desire, arousal, and orgasm), most of the participants reported no changes with respect to the pre-lockdown period. For both sexual desire (69.7% men, 54.9% women) and arousal (72.7% men, 58.2% women), it was mostly the men who said that they did not notice any difference, while for orgasm, there was hardly any difference between the genders (78.8% men, 79.1% women). Finally, 9.1% of the men and 26.4% of the women declared that their frequency of sexual intercourse had increased during the lockdown, whereas 24.2% of the men and 30.8% of the women reported a decrease in frequency. These results show that there may be gender differences in the way the lockdown influenced the frequency of sexual intercourse, seemingly because that aspect of sexuality is more easily influenced in women. These conclusions are further confirmed by the fact that more men than women answered that they perceived no differences at all (76.7% men, 42.9% women), as depicted in Figure 1. With regard to the increase in sexual desire, arousal, orgasm, and frequency, the answers were very much similar for all these items and can be summarized in some macro-categories, such as more free time, more time with the partner, less stress, and boredom. Some examples of the received answers are: “more serenity, less stress and more time to think about oneself,” “availability of more free-time,” “more rest and consequently more energy,” “boredom and greater closeness to the partner,” “desire to be together and take advantage of the time for doing something pleasurable,” and “we both felt less stressed working from home and therefore, more serene and open to our relationship.” In relation to the decrease in the aforementioned items, the summarized macrocategories are more stress, forced coliving, routine, anxiety and preoccupation about the job, anxious and worrisome feelings about the situation, feeling the partner’s distance, and absence of privacy. Some examples of the collected answers are “seeing each other 24 × 7,” “feeling of forced imprisonment and absence of freedom,” “absence of privacy,” “excessive workload,” “constant intimacy,” “not being able to relax,” and “the transition from living separately to always being together.”

This study also investigated the other aspects of a couple’s sexuality such as pleasure, the importance of sexuality, satisfaction, perception of the partner’s satisfaction, and reaction to the partner’s sexual advances. Regarding sexual satisfaction, 3% of the men and 13.3% of the women indicated an increase, while 6.1% of the men and 15.4% of the women reported a decrease. For the item about the perception of the partner’s satisfaction, 3% men and 16.5% women reported an increase in this perception, while 6.1% men and 15.4% women indicated a decrease. It is notable that for almost all the men, there were no changes in any of the aspects considered, while there were some changes for women. This finding was true for all the areas mentioned previously, and in particular, 90.9% of the men and 71.4% of the women reported no change in sexual satisfaction, whereas 90.9% of the men and 68.1% of the women reported no change in the perception of their partner’s satisfaction.

Moreover, regarding their reaction to their partner’s sexual advances, none of the men reported a change, but 25.3% of the women reported a change. With the aim of giving participants some space to freely express themselves, they were again given the opportunity to answer an open-ended question about what changes they experienced while reacting to their partner’s advances. In response to the question, *“Why has sexuality changed?”* some of the participants indicated that there was an increase in acceptance of the advances, while the others reported a decrease.

At the end of the questionnaire, there were two open-ended questions about the changes in sexuality that participants may have experienced during the lockdown and the reasons behind these changes. The macrocategories identified to summarize these findings are the same ones that were previously used to report changes in sexual desire, arousal, orgasm, and frequency, as reported above. Some examples of the answers received are “more free time and better physical contact,” “stress and too much closeness 24 × 7,” “Although I feel that my desires have increased and my partner’s have decreased because of stress and routine, we talk about it freely and have sex almost every day,” “difficulty in diverting thoughts from anxieties and worries,” “more time to dedicate to sexuality as well as physical and emotional closeness as a couple,” “the children are always present and my wife is worried,” and “fear of contagion and stress related to the new restrictions.” According to the perceptions reported by the research participants, the lack of privacy and the constant closeness impacted sexual habits negatively, whereas participants who spent only a few hours together prior to the lockdown were enjoying the closeness and had seen an improvement in their sexual lives.

A cluster analysis based on the mentioned changes in sexuality has been performed. The analysis provided two groups: Cluster 1 (*N* = 57) is characterized by the higher number of changes in sexual life during COVID-19, Cluster 2 (*N* = 67), on the contrary, includes the participants who have perceived very few or no differences in sexuality. Cluster 1 was almost totally composed by women, as it significantly encompassed less men (*N* = 9, 27%; χ^2^ = 6.32, *p* < 0.01) than Cluster 2 (*N* = 24, 73%). Table 2 reports differences in the study variables between Cluster 1 and Cluster 2. The two clusters differed only for the personal variables, where participants in Cluster 1 showed higher feelings of depression (*t* = 2.60, *p* < 0.01), anxiety (*t* = 2.05, *p* < 0.05), and stress (*t* = 2.86, *p* < 0.01).

**TABLE 2:** Mean scores, standard deviation, and differences between clusters in the study variables.

	Cluster 1 (*N* = 57)		Cluster 2 (*N* = 67)			
	*M*	SD	*M*	SD	*t*	*p*
Age	33.04	8.39	34.86	8.50	–1.16	ns
DASS total score	17.61	11.05	12.54	8.76	2.85	<0.01
DASS depression	6.18	4.08	4.27	4.05	2.60	<0.01
DASS anxiety	3.26	3.47	2.24	2.15	2.05	<0.05
DASS stress	8.18	4.59	6.03	3.75	2.86	<0.01
SDI total score	46.70	12.67	4.66	2.89	–0.87	ns
SDI dyadic	32.12	7.29	32.96	7.99	–0.60	ns
SDI solitary	14.58	8.32	15.69	8.21	–0.74	ns
BISF autoerotism	2.55	1.39	2.90	1.33	–1.43	ns
BISF couple sexuality	3.44	1.10	3.55	0.72	–0.67	ns
PHQ15	5.00	3.87	4.66	2.89	0.57	ns
QMI	39.61	6.83	38.42	7.57	0.92	ns

Table 3 shows the results of logistic regression analysis on the couples’ quality of marriage during the lockdown in the research study. The model is significant for χ*^2^* = 16.60 (*p* < 0.05) and being older and feeling more anxiety predicts a decrease in the overall perceived quality of marriage. During the lockdown, the participating couples had not perceived significant differences relating to the frequency, importance, and satisfaction of sexual intercourse. Nonetheless, small differences were perceived in relation to orgasm and desire. The most changes in sexual desire were influenced by the DASS general score [χ*^2^* = 9.33, *p* < 0.01; *B* = 0.136 SE = 0.048, *OR* = 1.14 (1.044–1.26)], while changes in the quality of orgasm were influenced by somatic symptoms [PHQ-15, χ*^2^* = 9.50, *p* < 0.01; *B* = 0.189 SE = 0.09, *OR* = 1.21 (1.060–1.37)].

**TABLE 3:** Binary logistic regression analysis results with dependent variable Quality of Marriage Index cutoffs.

	B	SE	Wald	*p*	OR	95% C.I.	
						Low	High
Age	0.060	0.026	5.202	0.023	1.062	1.008	1.118
Gender	−0.381	0.488	0.608	0.436	0.683	0.262	1.779
DASS depression	0.146	0.086	2.900	0.089	1.157	0.978	1.368
DASS anxiety	−0.203	0.103	3.865	0.049	0.816	0.667	0.999
DASS stress	0.047	0.083	0.313	0.576	1.048	0.890	1.233
PHQ15	0.085	0.072	1.402	0.236	1.088	0.946	1.252

## Discussion

The present research aims at assessing preliminary changes in couple sexuality during the first few weeks of the COVID-19 lockdown in Italy. Following the suggestion of Ahmed et al. (2020), we administered an online survey to investigate possible changes in couples’ sex lives. Our first result was unexpected but very interesting: despite the psychological consequences of this challenging situation, when asked directly, most of the couples did not perceive any differences in their sexuality. Other studies on sexuality during the lockdown considered the frequency of sexual activity or sexual intercourses. In the United Kingdom and Spain, respectively, only 41 and 39% of the participants had maintained the same frequency (Ibarra et al., 2020); decrease in sexual intercourse frequency was found in 60% of United Kingdom participants (Jacob et al., 2020), and a decrease in sexual activity was found in 37% of the Chinese participants (Li G. et al., 2020). In three south-east Asian countries, nearly 70% of the participants engaged in sexual activity with their partner one to five times a week or more, with a considerable increase from before the lockdown (Arafat et al., 2020); similar results were found in Turkish women (Yuksel and Ozgor, 2020). A study on 1,515 young Italians found that, although most of the participants experienced an increase in sexual desire and arousal, this did not translate into an increase in the frequency of sexual intercourse (Cocci et al., 2020).

In our study, some female participants reported a decrease in pleasure, satisfaction, desire, and arousal (Figure 1). According to their open-ended answers, worry, lack of privacy, and stress appear to be the main reasons for the changes in women’s sexuality, especially the decrease in their excitation and quality of pleasure. These data are in line with various studies, such as that of Li S. et al. (2020), in which 39% of the women reported a decrease in sexual satisfaction and the quality of sexual activity; that of Cocci et al. (2020), where 53.3% of the participants perceived less satisfaction in sexual relations compared to the pre-quarantine period; and Yuksel and Ozgor’s (2020) study that reported a significant decrease in arousal, satisfaction, and difficulty in reaching orgasm. Moreover, although male and female participants reported few differences in their sexual life during the COVID-19 lockdown, gender differences were confirmed by the cluster analysis, showing that the group reporting the more changes was mostly composed by women. In addition, as reported in Table 2, based on the changes in sexuality, a part of the sample (Cluster 1) was more subject to modifications than the other. The *t*-test analysis showed that participants in Cluster 1 experienced more negative feelings, such as anxiety, depression, and stress. It can be argued that personal emotions and psychological difficulties during the lockdown had an impact on participants’ sexual life, more than specific aspects related to the couple’s relationship. Moreover, previous studies indicated that stress, anxiety, and depression have a negative impact, especially on women’s sexuality (Dèttore et al., 2013; Kalmbach et al., 2014). In our sample, the decrease in sexual desire is mostly influenced by the DASS total score based on anxiety, stress, and depressive symptoms; also, Cocci’s study confirmed that women, more frequently than men, experienced anxiety and depressive symptoms (Cocci et al., 2020). These data are in line with previous literature suggesting that a pattern of negative emotional symptoms can lead to a lower level of sexual desire (Rosen et al., 2009; Worsley et al., 2017).

Finally, aging and anxiety are also responsible for decreasing relationship satisfaction (Table 3). This result can be explained by the answers given by the participants themselves: on the one hand, for older participants, spending all the time with children and family can result in a lack of privacy with fewer moments of intimacy with the partner. On the other hand, fear of COVID-19 infection and the prolonged lockdown have generated higher levels of anxiety in participants, leading to worsening of their relationship satisfaction. Another possible explanation is that older participants not only are concerned for their safety but also the safety of their offspring, and they are carrying the burden of children management and homeschooling. Additionally, the lockdown has forced couples to social isolation, and parents had to face their daily life without the help of nannies and grandparents. The anxiety connected to these specific aspects, as well as the uncertainty for the future, negatively affected the couple’s relationship, since the partner was the most probable and only person with whom to confront and lean on. Future studies should assess the outcomes of persistent somatic symptoms due to the COVID-19 lockdown on sexuality, after the emergency period.

## Limitations

The present research has a few limitations that need to be addressed. In the first place, the number of participants was quite low due to the taboos and difficulties to source people who would be willing to divulge about their sexual life, especially during a time of stress. Second, since the cross-sectional methodology does not allow causal connections between the study variables, longitudinal studies should be carried out. Finally, our sample was composed mainly of heterosexual participants, whereas a convenience sample of LGBTQA+ (lesbian, gay, bisexual, transgender, intersex, queer/questioning, asexual, and many other terms such as non-binary and pansexual) should also be investigated to assess the relation between COVID-19 lockdown and sexuality, and to provide more specific results.

## Conclusion

These preliminary data suggest that even if participants seem to show higher levels of resilience, the negative aspects of lockdown, such as anxiety, lack of privacy, fear of health conditions, and psychosomatic symptoms, can affect the quality of sexual life. The implications of these results are both clinical and research-related. In the first place, the main emotional and relational problems emerging from the survey will be used to conduct online focus groups on sexuality during the COVID-19 lockdown, with the purpose of addressing in a positive, warm, and non-judgmental environment how couples’ sexual life has changed and their main needs. Moreover, to better address patients’ needs and experiences, and provide *ad hoc* interventions, future clinical therapeutic approaches should take into consideration what has already emerged and what will eventually emerge from this research, on sexual well-being during this unprecedented time of crisis.

## Data Availability Statement

All datasets presented in this study are included in the article/Supplementary Material.

## Ethics Statement

The studies involving human participants were reviewed and approved by the Ethics Committee of Psychological Research Area 17 of Padua University. The patients/participants provided their online explicite informed consent to participate in this study.

## Author Contributions

MP and LF involved in all aspects of the research project: design, conducting the research, data handling, statistical analysis, drafting, and editing the manuscript. AC and RF assisted with the design of the study, in data collection and handling, and editing of the manuscript. All authors contributed to the article and approved the submitted version.

## Conflict of Interest

The authors declare that the research was conducted in the absence of any commercial or financial relationships that could be construed as a potential conflict of interest. The reviewer PR declared a past collaboration with one of the authors LF to the handling Editor AG.
